# Microbial Biofilm Community Variation in Flowing Habitats: Potential Utility as Bioindicators of Postmortem Submersion Intervals

**DOI:** 10.3390/microorganisms4010001

**Published:** 2016-01-04

**Authors:** Jennifer M. Lang, Racheal Erb, Jennifer L. Pechal, John R. Wallace, Ryan W. McEwan, Mark Eric Benbow

**Affiliations:** 1 Department of Biology, University of Dayton, Dayton, OH 45469-2320, USA; jenm1006@gmail.com (J.M.L.); ryan.mcewan@udayton.edu (R.W.M.); 2 Department of Biology, Millersville University, Millersville, PA 17551, USA; rnerb@iastate.edu (R.E.); John.Wallace@millersville.edu (J.R.W.); 3 Department of Entomology, Michigan State University, 243 Natural Science Building, 288 Farm Lane, East Lansing, MI 48824, USA; pechalje@msu.edu; 4 Department of Osteopathic Medical Specialties, Michigan State University, 243 Natural Science Building, 288 Farm Lane, East Lansing, MI 48824, USA

**Keywords:** epinecrotic, succession, epilithic, forensic, freshwater, necrobiome, ARISA (automated ribosomal intergenic spacer analysis), PMSI

## Abstract

Biofilms are a ubiquitous formation of microbial communities found on surfaces in aqueous environments. These structures have been investigated as biomonitoring indicators for stream heath, and here were used for the potential use in forensic sciences. Biofilm successional development has been proposed as a method to determine the postmortem submersion interval (PMSI) of remains because there are no standard methods for estimating the PMSI and biofilms are ubiquitous in aquatic habitats. We sought to compare the development of epinecrotic (biofilms on *Sus scrofa domesticus* carcasses) and epilithic (biofilms on unglazed ceramic tiles) communities in two small streams using bacterial automated ribosomal intergenic spacer analysis. Epinecrotic communities were significantly different from epilithic communities even though environmental factors associated with each stream location also had a significant influence on biofilm structure. All communities at both locations exhibited significant succession suggesting that changing communities throughout time is a general characteristic of stream biofilm communities. The implications resulting from this work are that epinecrotic communities have distinctive shifts at the first and second weeks, and therefore the potential to be used in forensic applications by associating successional changes with submersion time to estimate a PMSI. The influence of environmental factors, however, indicates the lack of a successional pattern with the same organisms and a focus on functional diversity may be more applicable in a forensic context.

## 1. Introduction

Understanding pattern and process in ecological community development is a long-established area of scientific inquiry that has new and important implications in microbial community ecology and microbial resource management. This includes the potential for utility as bioindicators of stream health or within the forensic sciences. The predictability of temporal organismal community development (succession) is a topic with a long-history of scientific debate [1,2], and linking community patterns to resources and disturbances across space and time has generated large bodies of theory and empirical results [3,4]. Using patterns of community succession and assembly have been applied in different contexts, and these include microbial ecology (e.g., biofilm formation) and the forensic sciences (e.g., forensic entomology) [5,6].

In aquatic ecosystems, microbes are found predominantly within biofilms rather than free floating within the water column [7,8]. Biofilms are matrix-enclosed microbial communities that are both trophically (heterotrophs and autotrophs) and phylogenetically (prokaryotes and eukaryotes) diverse. These microorganisms of algae, bacteria, fungi, and protozoa [8,9] are encased in an extracellular polymeric substance (EPS) that is protective against changing abiotic conditions [10,11], traps and stores nutrients [12,13,14], and accrues enzymes that break down organic matter [15,16]. The type of surface biofilms develop on classifies them, and this surface strongly influences community composition and energy dynamics. For example, epilithic biofilms are found on inorganic substrates (e.g., rocks) and are considered autotrophic due to the presence of an abundant algal community, assuming adequate light; whereas, epixylic biofilms that are found on decomposing plant matter, have a substantial fungal community, and are considered heterotrophic [17,18,19]. Epinecrotic biofilms can be considered heterotrophic because they form on an organic substrate [5], but light availability also allows substantial algal communities to develop [20,21]. Other environmental factors affect biofilms as well. For example, flow, nutrients, and water chemistry are important drivers of epilithic biofilm community assembly [22,23,24,25]. These factors should be considered for epinecrotic communities, or those microbial communities that are part of decomposing heterotrophically-derived biomass; however, little is known about the microbial community development of epinecrotic biofilms. To our knowledge, the role of environmental factors in influencing community composition of epinecrotic biofilms is an unexplored area of scientific research.

Patterns of diversity and abundance in epinecrotic biofilms throughout aquatic decomposition have been demonstrated as indicators of decomposition progress using diatoms in freshwater systems [21,26] and bacteria in a marine setting [27]. The potential for using succession was further described for bacteria in freshwater streams where pyrosequencing revealed changing communities in both summer and winter seasons; however, biofilm communities were more similar to each other within a season. This observation suggests that environmental factors influence composition more than the decomposition process [28]. Understanding how these communities assemble during succession and identifying how microbial diversity varies among substrate types (e.g., tiles *vs.* carrion) within a flowing habitat has the potential to reveal underlying mechanisms that can be applicable in areas of biofilm management and forensics.

The type of evidence collected at a death investigation scene greatly varies. Biological evidence using successional patterns of invertebrates, such as insects (e.g., flies and beetles), is an important tool for estimating a minimum post mortem interval (PMI_min_, the amount of time from death to discovery) in terrestrial settings [29]. This approach of using invertebrate succession, however, has been unsuccessful in estimating a postmortem submersion interval (PMSI, the time from submersion to discovery) in aquatic settings [30,31,32]. This is primarily because aquatic invertebrates typically use the remains as a substrate and not as an energy resource like the necrophagous terrestrial invertebrates. These insects have evolved to detect and use these sources of heterotrphically-derived biomass as a resource for reproduction [6,33], while aquatic insects have not. Yet, biofilms are ubiquitous, and the epinecrotic microbial communities have been noted on salmon [34], waterfowl [35], rats [36], and swine [20]. Utilizing these epinecrotic biofilms has been investigated as an alternative approach to measure PMSI [28] because biofilms develop in a successional manner where community composition changes over time. These compositional changes can be assessed using molecular sequencing approaches and used to associate microbial community changes with decomposition time [5,12,17].

The objective of this study was to assess microbial community development of epinecrotic biofilms under varying environmental conditions. Previous work has indicated clear evidence of successional patterns and environmental influence in epilithic biofilm communities [25], and in this study we sought to compare the response of epilithic and epinecrotic communities by assessing community composition through time and in relation to varying environmental conditions. We hypothesized (H_1_) that epinecrotic and epilithic biofilms would have different biofilm community composition regardless of differences in environmental factors, *i.e.*, resource substrate is a stronger ecological control than environmental conditions, such as water temperature. We further hypothesized (H_2_) that environmental factors would drive community composition within biofilms that developed on the same substrate type. Our last hypothesis (H_3_) was that both the epilithic and epinecrotic biofilms would exhibit community differentiation based on development time (succession), supporting previous research on biofilm communities in aquatic habitats. Epinecrotic communities were distinctly different from epilithic communities and environmental variability associated with geographic location also drove community composition. The biofilm communities also exhibited patterns of succession by changing over time supporting our hypotheses.

## 2. Methods

### 2.1. Study Site Descriptions

The experiment was conducted in two locations (Farmersville, OH and Millersville, PA, USA) to capture the effects of differing environmental factors related to geographic region. The Farmersville, OH study was conducted from 29 June 2012 to 27 July 2012 in a first order headwater stream that joined Little Twin Creek (39°39′53.8″ N 84°23′44.3″ W). The surrounding land cover of the catchment area was predominately agriculture, and there was a 2–5 m riparian forest buffer that was dominated by maple (*Acer* sp.), elm (*Ulmus* sp.), and Amur honeysuckle (*Lonicera maackii* (Rupr.) Maxim.). A drought eliminated flow to the upper portions of the stream and the study sites had to be relocated further downstream, which resulted in two different habitats that were intersected by a concrete divide under a bridge. The upstream site was shaded by trees (closed canopy), had clear flowing water, and the substrate was composed of pebbles and cobbles. The downstream site was subjected to direct sunlight (open canopy), had clouded almost stagnant water, and the substrate consisted of cobbles and pebbles that were covered by sediments. The upstream site was more similar to the Millersville, PA site based on these described features than to the downstream site.

The Millersville, PA study site has been previously described by Benbow *et al.* [28], and this study was conducted from 26 June 2012 to 17 July 2012 in a first order tributary to the west branch of Big Spring Run (39°59′29.1″ N 76°15′49.0″ W) within the Conestoga River watershed of Lancaster, PA, USA. The watershed is a mix of suburban/agricultural land, and the stream was bordered by 10–20 m of riparian forest buffer and shrub vegetation. The riparian forest was dominated by silver maple (*Acer saccharinum* L.), box elder (*Acer negundo* L.), and sycamore (*Platanus occidentalis* L.) with a ground cover of multiflora rose (*Rosa multiflora* Thunb.). The stream substrate consisted of a mixture of pebble and cobble.

### 2.2. Environmental Water Parameters

In Farmersville, OH, USA specific conductivity (SpCond μS/cm), total dissolved solids (TDS mg/L), pH, and temperature (°C) were recorded using a YSI 6600 v2 Sonde (YSI Inc., Yellow Springs, OH, USA) 15 m above and below the uppermost and lowermost carcasses, respectively. Water was also collected at these points to measure nitrate (mg/L NO^3−^-N), nitrite (mg/L NO_2_^−^-N), sulfate (mg/L), ammonia (mg/L NH_3_-N), alkalinity (mg/L CaCO_3_), and total suspended solids (TSS mg/L) in the lab using EPA approved protocols (Hach^®^ Company, Loveland, CO, USA). In Millersville, PA, USA water quality parameters of dissolved oxygen (mg/L), pH, specific conductivity (μS/cm), water temperature (°C), total dissolved solids (g/L), oxidation reduction potential (mV), and salinity (ppt) were measured at a single location 30 m upstream of the uppermost carcass and 30 m downstream of the lowermost carcass on each sampling day using a Horiba^®^ (Kyoto, Japan) Multi Water Quality Checker (U-50 Series).

### 2.3. Epinecrotic Biofilm Study Design and Sampling

Epinecrotic biofilms were allowed to develop on stillborn and submerged *Sus scrofa domesticus* carcasses (*n* = 4 per geographic region) obtained from the Penn State University Swine Research Facility (State College, PA, USA) as described in detail in a companion study [28]. Here, we summarize these methods. Swine skin has been accepted as a surrogate for human skin [37,38,39] and swine carcasses are frequently used in place of human cadavers [40] in forensic science research [41,42]. Carcasses were placed on plastic drawer organizer trays (0.38 m × 0.15 m × 0.05 m) inside metal small game traps (Havahart^®^, Animals B-Gone, Orrstown, PA, USA) (0.61 m × 0.18 m × 0.18 m) to facilitate sampling as the carcass disarticulated and to prevent large scavenger (e.g., raccoon) removal of the carcasses. Traps were anchored to the streambed on previously secured rebar, which allowed easy trap removal for sampling. Carcasses were placed ~15 m downstream from each other in run habitats to avoid being silted over in pool habitats and eliminate the abrasive action of riffle habitats.

Epinecrotic biofilms were sampled, while taking caution not to sample the same areas and to not repeat collections, using individually packaged sterile swabs before being placed into streams (Day 0) and then bi-weekly alternating 3 or 4 days from the abdomen/rib cage. The area was swabbed with six strokes where the swab was rotated 180° after the third stroke and one direction counted as a stroke. The swabs ware placed individually into sterile microfuge tubes and transported on ice and kept at −20 °C until DNA extraction. Carcasses were immediately submerged upon sample completion, and new gloves were worn for each carcass sampling event.

### 2.4. Epilithic Biofilm Study Design and Processing

Epilithic biofilms developed naturally on hexagonal unglazed porcelain tiles (*n* = 6) attached to brick pavers (19.2 cm × 9 cm × 1.3 cm) with 100% silicone. Pavers (*n* = 4) were placed 0.3 m upstream of the cages and immediately downstream of the cages. Tiles (*n* = 4, one from each paver) were removed at each sampling date, transported to the lab on ice, and placed at −20 °C until processing. Biofilm biomass was removed using a sterile razor blade and disposable interdental toothbrush and suspended in ultrapure water (NANOpure II; Barnstead, Boston, MA, USA). Two subsamples were collected on GB-140 glass membrane filters (diameter, 25 mm; pore size, 0.4 μm; Sterlitech, Kent, WA, USA) to determine total biomass as ash free dry mass (AFDM) and algal-associated biomass as chlorophyll *a* following established techniques [43]. A third subsample was collected for DNA extractions, and these filters were stored in 90% ethanol at −80 °C until extraction. Although for DNA extractions, we highly suggest using a mixed nitrocellulose filter and placing a few drops (2–3) of ethanol (96%–100%) on the sample that evaporates before storage.

### 2.5. DNA Extraction

DNA was extracted from ethanol evaporated, dried filters and swabs using a combination of methods [44,45] as suggested by others [46] and using methods of our previous aquatic biofilm research [25]. Samples were lysed in 1 mL extraction buffer (100 mM Tris-HCl (pH 8.0), 100 mM EDTA disodium salt (pH 8.0), 100 mM sodium phosphate (pH 8.0), 1.5 M sodium chloride and 1% CTAB (cetyltrimethyl ammonium bromide), 20 μL of proteinase K (10mg/mL), and 25 μL of SDS (sodium dodecyl sulphate) (20%) using bead beating (0.25 g each of 0.1 mm and 0.5 mm glass beads) for 15 min on a horizontal vortex adaptor (MO BIO Laboratories, Carlsbad, CA, USA) at full speed. Then, the samples were incubated at 60 °C for 30 min with gentle end-over-end inversions by hand at the midpoint of 15 min; 750 μL of supernatant for epilithic samples and 250 μL for epinecrotic samples was collected in a new microcentrifuge tube after centrifugation at 6000× *g* for 10 min. For epinecrotic swab samples only, the lysis process was repeated without vortexing with an additional 500 μL extraction buffer, 10 μL proteinase K, and 12.5 μL SDS to obtain a final supernatant volume of approximately 750 μL. The protocol was adjusted to maximize extraction from the different sample types, but one extraction should be sufficient. DNA was separated from organic debris with a chloroform: isoamyl alcohol (24:1 *v*/*v*) extraction and precipitated overnight at −20 °C using isopropanol. Samples were removed from the −20 °C and warmed to 37 °C to dissolve salt precipitates, and the DNA was pelleted at 15,000× *g* for 30 min. Finally, the DNA pellet was washed twice with ice cold 70% ethanol and dissolved in 50–100 μL ultrapure water (NANOpure II™, Thermo Scientific, Waltham, MA, USA) water, depending on the DNA pellet size.

### 2.6. ARISA

Bacterial communities were assessed using profiles created by automated ribosomal intergenic spacer analysis (ARISA) [47]. The ARISA approach generates a unique “fingerprint” of microbial communities using the 16S-23S intergenic space in bacteria. While ARISA does not taxonomically identify organisms like sequencing methods, this method generates community profiles that produce similar patterns within results [48,49]. Approximately 15–20 ng of DNA quantified by spectrophotometer (NanoPhotometerTM Pearl; Denville Scientific Inc., South Plainfield, NJ, USA) was amplified by PCR using 25 μL GoTaq^®^ Colorless Master Mix (Promega, Madison, WI, USA) with 0.5 μM of forward and reverse primers. Bacteria ribosomal intergenic space regions were amplified with primers ITSF (5′-GTCGTAACAAGGTAGCCGTA-3′) labeled with FAM at the 5′ end (IDT, Coralville, IA, USA) and ITSReub (5′-GCCAAGGCATCCACC-3′) [50]. Fragments were created with the following PCR conditions: (i) 94 °C for 3 min; (ii) 35 cycles of 94 °C for 1 min, 56 °C for 1 min, 72 °C for 2 min; and finally (iii) 72 °C for 10 min [51]. PCR products were sent to DNA Analysis, LLC (Cincinnati, OH, USA) for fragment analysis on an ABI 3100 (Life Technologies, Carlsbad, CA, USA). Fragments were interpreted using Genescan v 3.7 using the Local Southern Size Calling Method with a peak height threshold of 100 fluorescence units to remove background fluorescence and formulated using GeneMapper v 2.5 (Life Technologies, Carlsbad, CA, USA).

Fragment peak length and area was converted to column format using the treeflap Excel Macro [52,53] and processed with the automatic_binner script to determine binning window size and the interactive_binner script to determine the best starting window position [54] in R v 3.1.0 (R Core Team 2014). This method was used to account for inherent imprecision of analyzer machines. Peak area was converted to relative abundance of each fragment as part of the entire sample, fragments < 0.09% relative abundance were removed [54], and window size was calculated to be 1.5 base pairs.

### 2.7. Statistical Analyses

Environmental water parameters throughout the study within a site were averaged and compared using a student’s *t*-test. Values were also compared within the Farmersville, OH site to compare the upstream/closed canopy habitat to the downstream/open habitat.

Microbial community patterns were visualized using nonmetric multidimensional scaling (NMDS) with Bray-Curtis (Sørensen) distance as we have done previously for both epinecrotic [28,41,42] and epilithic biofilm [25] community analyses because it is a nonparametric approach useful in evaluating nonlinear relationships of data with high numbers of zeros [55]. Differences in configuration based on categorical overlays were tested with nonparametric multivariate analysis of variance (PERMANOVA) to identify main and interacting effects, followed PERMANOVA post-hoc comparisons with Bonferroni corrections were used to further identify which days were significantly different from each other [56]. Because Bonferroni adjustments to *p*-values can be highly conservative, we also binned days into broader time frames for post-hoc comparisons. These binning was based on visual patterns in the ordinations To account for the conservative nature of Bonferroni corrections, we also evaluated the post-hoc comparisons with Holm corrections, which is less conservative and the results were the same. Analyses were conducted in R v 3.1.2 (R Core Team 2014) with the vegan v 2.2-1 package [57].

## 3. Results

### 3.1. Environmental Water Quality Characteristics

The study sites, including the upstream and downstream sites in Farmersville, OH, differed in water quality characteristics. Between Farmersville, OH, USA (Table 1) and Millersville, PA, USA (Table 2), there were only a few common parameters measured due to access of different measurement devices. Temperature was the only non-significant (*p* = 0.8546) parameter while dissolved oxygen (*p* = 0.0347), pH (*p* = 0.0091), specific conductivity (*p* < 0.0001), and total dissolved solids (*p* = 0.0066) were significantly different between the two geographic locations. Within Farmersville, OH, USA the upstream and downstream sites significantly differed in total suspended solids (*p* = 0.0178), specific conductivity (*p* = 0.0004), total dissolved solids (*p* = 0.0003), dissolved oxygen (*p* = 0.0008), pH (*p* < 0.0001), and temperature (*p* < 0.0001).

### 3.2. Microbial Biofilm Communities

**Figure 1:**
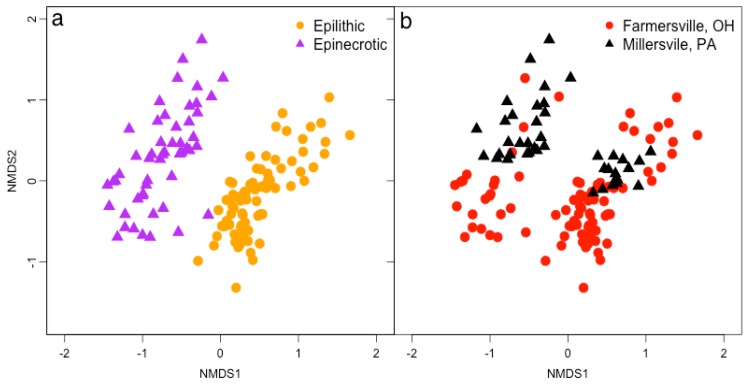
Bacteria community structure was visualized using nonmetric multidimensional scaling (3-D, *R*^2^ = 0.97, stress = 0.18) and overlaid with (**a**) biofilm type and (**b**) location of study and both factors were significant determined by PERMANOVA.

**Figure 2:**
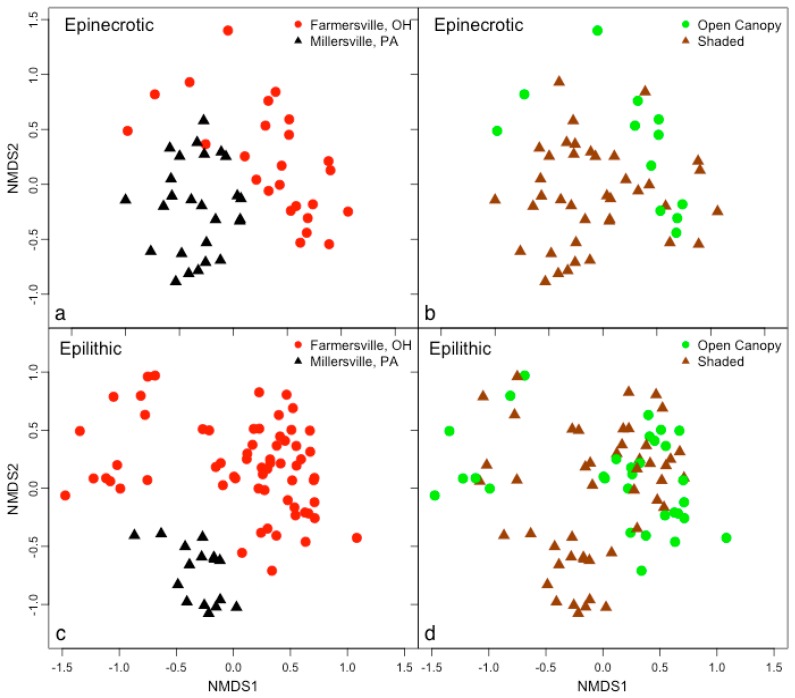
Bacteria communities were separated into (**a**) and (**b**) epinecrotic (3-D, *R*^2^ = 0.97, stress = 0.17) and (**c**) and (**d**) epilithic (3-D, *R*^2^ = 0.98, stress = 0.14) biofilms and community structure was visualized using nonmetric multidimensional scaling. (**a**) and (**c**) Location of study significantly influenced community structure of both biofilm types as determined by PERMANOVA, but only epilithic biofilms were significantly affected by (**b**) and (**d**) canopy.

Microbial community profiles were influenced by location and substrate. Epinecrotic and epilithic biofilms were clearly and significantly different (Figure 1; PERMANOVA, *pseudo-F* = 9.31, *p* < 0.0001), demonstrating that substrate was an important factor influencing community assembly. Location was also a significant factor (PERMANOVA, *pseudo-F* = 17.31, *p* < 0.0001), and there was a substrate × location interaction effect (PERMANOVA, *pseudo-F* = 7.02, *p* < 0.0001). When analyses were separated by substrate, *i.e.*, performed using only epilithic or epinecrotic data, location formed clear community clusters (left panels, Figure 2; PERMANOVA, *pseudo-F* = 4.68, *p* < 0.0001 (epilithic); *pseudo-F* = 11.46, *p* < 0.0001 (epinecrotic). The influence of site differences between the upstream/closed canopy and downstream/open canopy sites in Farmersville, OH was statistically significant for epilithic biofilms (PERMANOVA, *pseudo-F* = 3.66, *p* = 0.0002) but not epinecrotic biofilms (PERMANOVA, *pseudo-F* = 1.07, *p* = 0.3516). Substrate appeared to be the most influential factor on biofilm formation; however, location differences clearly influenced biofilms when epinecrotic and epilithic biofilms were separated in the analyses.

**Table 1:** Water quality parameters in Farmersville, OH, USA were measured when substrates were deployed and at every sampling date 15 m above and below the uppermost and lowermost carcasses, respectively. Nitrate (mg/L NO^3−^-N), nitrite (mg/L NO_2_^−^-N), sulfate (SO4^2−^ mg/L), ammonia (mg/L NH_3_-N), alkalinity (mg/L CaCO_3_), and total suspended solids (TSS mg/L) were measured in the lab using EPA approved protocols (Hach^®^ Company, Loveland, CO, USA). Specific conductivity (SpCond μS/cm), total dissolved solids (TDS mg/L), dissolved oxygen (DO mg/L), pH, and temperature (°C) were recorded using an YSI 6600 v2 Sonde (YSI Inc., Yellow Springs, OH, USA). Bolded means denote significant differences (*p* < 0.05) between the upstream and downstream sites using a two tailed *t*-test.

Date	Time (Days)	NO^3−^	NO_2_	SO4^2−^	NH_3_	CaCO_3_	TSS	SpCond	TDS	DO	pH	Temp
29 June	0	2.35	0.054	21.0	0.10	291	26	617	0.40	3.78	7.92	20.6
02 July	3	2.95	0.025	20.5	0.09	295	10	636	0.41	5.56	7.99	21.3
06 July	7	2.40	0.049	22.0	0.22	332	20	652	0.42	3.21	7.91	21.5
09 July	10	2.55	0.016	23.0	0.13	294	10	652	0.42	5.28	8.02	21.8
13 July	14	3.40	0.034	23.0	0.26	307	-	650	0.42	4.57	8.17	18.6
16 July	17	2.30	0.020	23.5	0.18	335	27	644	0.42	3.65	8.01	21.6
20 July	21	1.95	0.023	27.0	0.15	308	20	644	0.42	2.84	7.96	21.5
23 July	24	1.95	0.019	27.0	0.14	295	28	653	0.43	3.68	8.04	21.3
Mean	-	2.48 ± 0.49	0.030 ± 0.014	23 ± 2	0.16 ± 0.06	307 ± 17	20 ± 7	643 ± 12	0.42 ± 0.01	4.07 ± 0.97	8.00 ± 0.08	21.0 ± 1.1
Upstream/Shaded	-	2.39 ± 0.66	0.034 ± 0.024	24 ± 4	0.14 ± 0.05	313 ± 22	15 ± 6	666 ± 25	0.43 ± 0.01	2.87 ± 0.96	7.85 ± 0.10	19.7 ± 1.4
Downstream/Open	-	2.58 ± 0.67	0.025 ± 0.020	22 ± 1	0.17 ± 0.11	302 ± 18	34 ± 19	621 ± 13	0.40 ± 0.01	5.27 ± 1.36	8.16 ± 0.08	22.3 ± 0.7

**Table 2:** Water quality parameters in Millersville, PA of dissolved oxygen (mg/L), pH, specific conductivity (μS/cm), water temperature (°C), total dissolved solids (g/L), oxidation reduction potential (mV), and salinity (ppt) were measured at a single location 30 m upstream of the uppermost carcass and 30 m downstream of the lowermost carcass on each sampling day using a Horiba^®^ (Kyoto, Japan) Multi Water Quality Checker (U-50 Series).

Date	Time (Days)	DO	SpCond	ORP	pH	Salinity	TDS	Temperature
26 June	0	6.94	966	93.0	8.2	0.48	0.483	18.3
29 June	3	4.00	928	64.5	8.1	0.46	0.464	20.5
03 July	7	6.80	1038	66.8	8.5	0.52	0.519	20.1
06 July	10	6.55	950	47.8	8.2	0.47	0.475	20.7
10 July	14	4.05	908	37.2	8.1	0.45	0.454	22.2
13 July	17	4.34	1130	37.9	8.1	0.57	0.565	20.1
17 July	21	4.78	963	36.7	8.1	0.48	0.459	23.5
20 July	24	5.59	779	29.6	8.1	0.38	0.389	21.8
Mean	-	5.38 ± 2.25	957 ± 101	51.7 ± 21.5	8.2 ± 0.1	0.47 ± 0.05	0.476 ± 0.051	20.9 ± 1.6

**Figure 3:**
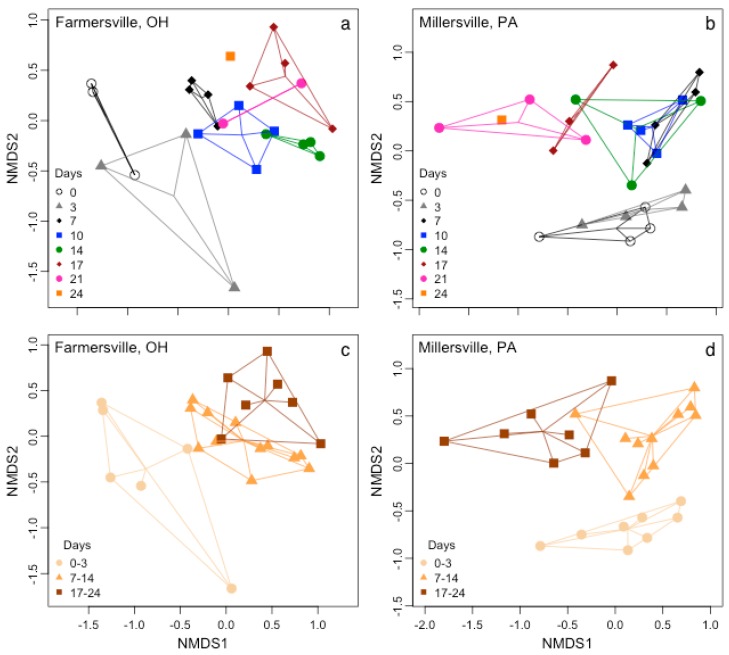
Succession of (**a**) and (**c**) Farmersville, OH (3-D, *R*^2^ = 0.98, stress = 0.14) and (**b**) and (**d**) Millersville, PA (3-D, *R*^2^ = 0.99, stress = 0.11) epinecrotic biofilms were depicted with NMDS ordination using the (**a**) and (**b**) days of decomposition and (**c**) and (**d**) binned categories as an overly with convex hulls and spider lines that connect at the group midpoint. Groups for both communities were significantly different as determined by PERMANOVA.

Successional patterns in epinecrotic biofilms were evident in ordination space, shown by the communities aligning in a chronological order at both locations (Figure 3). This time effect was significant in both Farmersville, OH (*pseudo-F* = 3.16, *p* < 0.0001; Figure 3) and Millersville, PA, USA (*pseudo-F* = 3.93, *p* < 0.0001; Figure 3). Riparian forest canopy did not have a significant effect on epinecrotic biofilms in Farmersville, OH (*pseudo-F* = 1.02, *p* = 0.3933) indicating robustness against environmental variability (Table 1) within the same geographical location. These data suggest that epinecrotic biofilm succession was not influenced by local environmental variation. There were several days determined to be significantly different when using Bonferroni corrected post-hoc comparisons (Table 3). The two comparisons that were consistent between the two locations were 0 *vs.* 7 days and 7 *vs.* 17 days. Days of decomposition were further binned into three time frames based on visual patterns observed in the NMDS ordination (Figure 3), and all comparisons were significantly different (Table 3). There were significant shifts in community composition between 3 and 7 days and then again between 14 and 17 days.

**Table 3:** Differences in communities based on day of decomposition were determined using post-hoc comparisons with Bonferroni corrections. Days were further binned into three timeframe categories based on visual patterns to further comparisons in Figure 3.

Day	Dayton		Millersville	
	*Pseudo-F*	*p*-Value	*Pseudo-F*	*p*-Value
0 *vs.* 3	1.7683	**0.0292**	1.5209	0.8925
0 *vs.* 7	2.0327	**0.0042**	2.1454	**0.0441**
0 *vs.* 10	2.7234	0.6000	2.2779	**0.0336**
0 *vs.* 14	4.2148	0.6000	3.6802	0.1000
0 *vs.* 17	3.4430	0.6000	2.6717	0.1500
0 *vs.* 21	0.0286	1.0000	1.8198	0.5042
3 *vs.* 7	1.3976	1.0000	1.7396	**0.0021**
3 *vs.* 10	1.6904	1.0000	1.9337	**0.0357**
3 *vs.* 14	2.8101	0.6000	3.2167	**0.0333**
3 *vs.* 17	2.2252	**0.0042**	2.1349	0.1000
3 *vs.* 21	1.2239	1.0000	1.4336	1.0000
7 *vs.* 10	1.2510	1.0000	1.6097	1.0000
7 *vs.* 14	2.5020	**0.0021**	2.9106	0.1333
7 *vs.* 17	2.0530	**0.0021**	2.1186	**0.0042**
7 *vs.* 21	1.1205	1.0000	1.4207	0.2375
10 *vs.* 14	0.9974	1.0000	2.8144	0.2000
10 *vs.* 17	1.8117	1.0000	1.8826	0.6999
10 *vs.* 21	1.0865	1.0000	1.1429	1.0000
14 *vs.* 17	2.2648	0.6468	2.1364	1.0000
14 *vs.* 21	1.6193	1.0000	2.4703	0.3793
17 *vs.* 21	0.7587	1.0000	0.7478	1.0000
0–3 *vs.* 7–14	2.9224	**0.0018**	4.4026	**0.0003**
0–3 *vs.* 17–24	3.0783	**0.0012**	4.2985	**0.0006**
7–14 *vs.* 17–24	2.4225	**0.0087**	3.2061	**0.0006**

**Figure 4:**
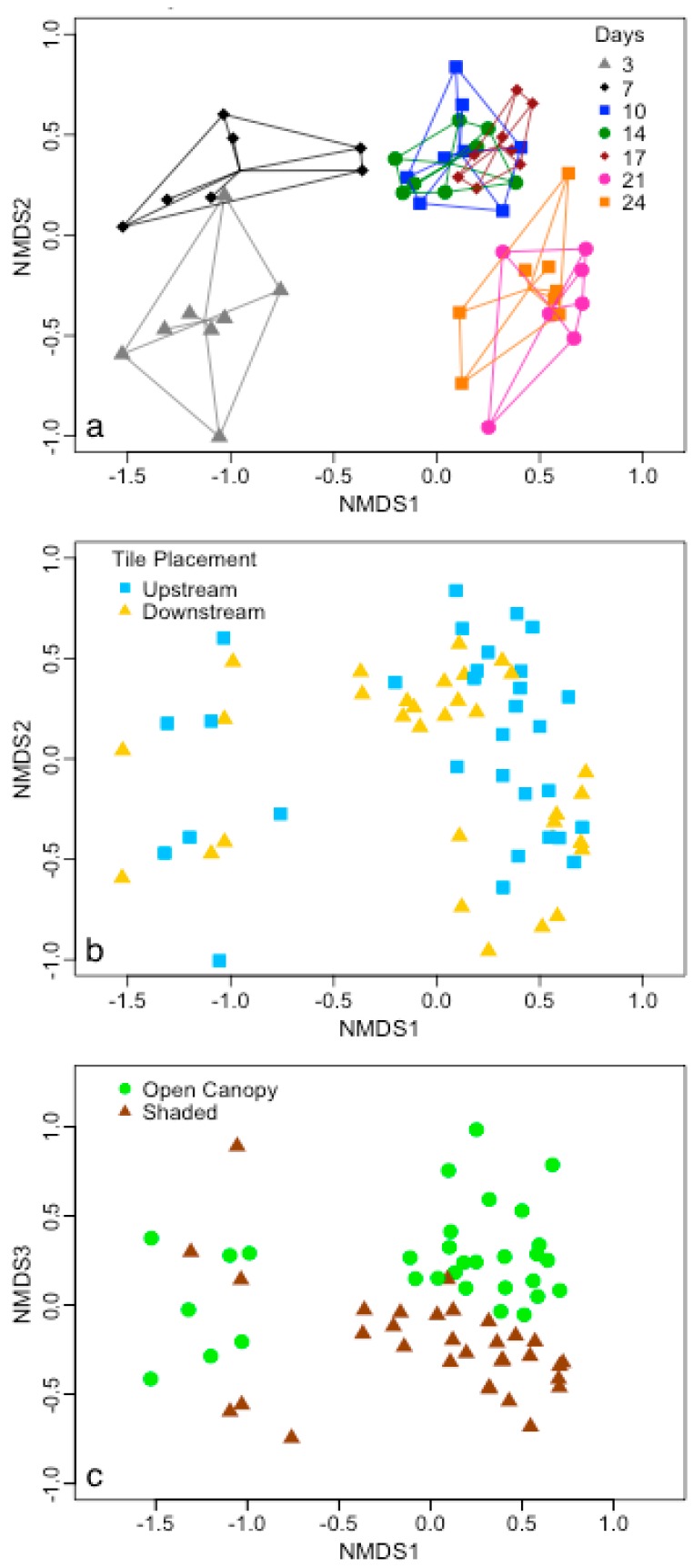
Bacterial community structure of epilithic biofilms in Farmersville, OH, USA is depicted using NMDS ordination (3-D, *R*^2^ = 0.98, stress = 0.13) and overlaid with (**a**) days of growth, (**b**) tile placement, and (**c**) canopy with convex hulls and spider lines that connect at the group midpoint. Both days and canopy were significant determined by PERMANOVA. Note that canopy ordinates along the NMDS Axis 3.

Epilithic biofilms were further analyzed to provide a reference to epinecrotic biofilms and to determine if carcass decomposition influenced the surrounding microbial community during colonization compared to an inorganic control. Only Farmersville, OH epilithic biofilms were analyzed because the Millersville, PA, USA biofilms had a low sample size (*n* = 16), only three sample dates had successful reactions, and the data had significantly different group dispersions (ANOVA, *p* = 0.0225). Epilithic biofilms were influenced by days of decomposition (*pseudo-F* = 12.40, *p* < 0.0001) and forest canopy (*pseudo-F* = 4.15, *p* < 0.0001), which had an interaction effect (*pseudo-F* = 2.31, *p* = 0.0093; Figure 4). It is interesting to note that forest canopy aligned with NMDS Axis 3 (Figure 4), but the effect was not observed until 10 days since communities of day 3 and 7 align with lower values of NMDS Axis 1. It is not surprising that light availability induced differences in bacteria at this time point because algae tend to become dominate later during epilithic biofilm succession and there is a strong algal-bacterial relationship within these communities [25]. The result also highlights sensitivity differences between epilithic and epinecrotic biofilms because there was no effect of forest canopy on epinecrotic biofilms; thus, indicating that epinecrotic biofilms were less sensitive than epilithic biofilms to environmental variability. Whether epilithic biofilms were placed upstream or downstream of the swine carcasses had no effect (*pseudo-F* = 1.61, *p* = 0.0695), which suggests that the carrion resource was not affecting the immediate surrounding environment in a way that altered epilithic biofilms.

## 4. Discussion

### 4.1. Substrate Type Affects Microbial Biofilm Communities

The colonization and development of microbial communities are known to be linked to resource substrates [18,22]. One objective in this study was to determine community differences of epinecrotic biofilms in relation to epilithic biofilms, the latter of which are virtually ubiquitous in streams. Substrate type (inorganic *vs.* carrion) demonstrated clear and significant differences in community composition between the two biofilm types. This finding was not entirely surprising because previous research has demonstrated that epilithic and epixylic (decaying plant material) biofilms differ in community composition, limiting nutrients, exoenzyme activity, and fungal biomass [17,18,58]. There were also functional differences because primary productivity was found to be higher in epilithic biofilms while respiration was higher in epixylic biofilms, which reflects differences in community composition [59]. It was beyond the scope of this study to identify the constituent organisms driving the separation of the epinecrotic and epilithic microbial communities; however, microorganisms dominating the epinecrotic community likely include a large portion of heterotrophs or detritivores whereas the epilithic community is likely represented by a larger portion of autotrophs. This difference may be why forest canopy cover had an influence on epilithic but not epinecrotic biofilms. Fungal organisms are an important component in structuring biofilms on plant derived organic substrates [60], and they may be important on carrion substrates as well. This biofilm component has yet to be studied in either terrestrial or aquatic epinecrotic biofilms and may provide a foundation for future studies. Because of the differences in community composition between biofilms that have colonized different substrates, our results provide initial, baseline information for potential future investigation into microbial profiling for forensic applications and other possible biofilm management opportunities.

### 4.2. Epinecrotic Community Response to Environmental Conditions

Ecological communities are known to vary in accordance with environmental conditions, yet, little is known about these effects in relation to epinecrotic biofilms. We hypothesized that within each biofilm type the environmental factors would influence biofilm community colonization and succession. The study locations (Farmersville, OH, USA and Millersville, PA, USA) were geographically distant from one another (*ca.* 800 km) and the communities clustered by location reflecting environmental differences. Dissolved oxygen, specific conductivity, and total dissolved solids were significantly different between the sites, and it is likely other unmeasured parameters (e.g., pH, nutrients) were different as well [61]. For instance, dissolved nutrients are readily utilized by biofilms [62] and available nutrients are known to influence biofilm community structure [63]. There may also have been variation among the sites in dissolved organic matter (DOM), which can select for specific bacterial communities even when source communities are different [22]. The differences observed in community composition could be related to geological substrate, land use history, or variation in the species pool, but most likely are a cumulative result of the environmental factors. It is important to note that local differences between the upstream/closed canopy and downstream/open canopy sites in Farmersville, OH, USA did not significantly influence epinecrotic communities, which implies that these biofilms were robust to some local environmental variability. Because the microbial communities change with some environmental conditions, these results suggest that the potential use of biofilm communities for stream health monitoring may have promise but depends on location and possibly season. Therefore, additional, large scale studies will be needed to more thoroughly understand how these communities vary in both geographic space and time.

### 4.3. Biofilm Community Succession

Succession is a foundational process in most ecosystems and has been clearly demonstrated in microbial communities [5]. We hypothesized that discernable variation over time would be found in the epinecrotic and epilithic biofilm communities. Indeed for all individual biofilm combinations (Figure 3 and Figure 4), community separation was clear across the study timeframe, but this was only visible when analyses excluded differences in location and substrate type. These findings build upon previous research showing that epilithic biofilm succession varies with flow [64], acidic conditions [24,65], and across seasons [9]. Within the context of epinecrotic biofilms, succession has been documented in a marine habitat [27] as well as a freshwater habitat during winter and summer [28]. The communities in freshwater were separated by season even though they developed in the exact same location [28]. Changing environmental factors in the same location has also been demonstrated in epilithic biofilms because resources exposed to light became more heterotrophic at the end of autumn [25]. While environmental conditions in our study were not significantly changing within location, they were different between locations. These data taken together with the literature highlights the overall importance of environmental conditions whether the comparison is temporal or spatial. Even with all the sources of environmental variability, succession occurred for both substrate types at each site, and this furthers the hypothesis that succession is a robust process that will occur across a broad range of conditions. Temporal resolution in our study was relatively coarse (days) and the communities were not always clearly separated (e.g., days 7, 10 and 14 were very similar in the Millersville, PA, USA site); however, we were able to identify shifts in communities at the first and second weeks of decomposition that were consistent in both locations. The shift was more pronounced between 3 and 7 days than it was between 14 and 17 days, which may reflect differences in successional rates. The community in the first shift could be transitioning from one derived from the mammal host to an environmentally based biofilm community, while the second shift may reflect successional stages within epinecrotic biofilms. This would result in the community turnover in the first shift being greater than in the second shift and could explain the observed degree of separation. Further supporting this idea, stark differences were seen between 0 days of decomposition and the next sampling date of 7 days at the genera and phylum level in a companion study [28]. These data suggest the possibility of developing a microbe-based timeline using more sensitive techniques for submerged bodies in forensic contexts. Additionally, by understanding successional changes in biofilm communities, this study also provides direction for future studies for using microbial communities within stream biofilms for biomonitoring purposes.

## 5. Conclusions and Potential for Applications and Management of Biofilms

Epinecrotic communities were distinctly different from epilithic communities regardless of the location indicating that these communities are unique. Selective forces of the substrate were greater than the influence of environmental factors between the two geographic locations; however, within biofilm type, environmental factors associated with geographic location drove community differences. All communities exhibited patterns of succession suggesting that this is a robust process that will consistently occur. The implications of this study are that epinecrotic communities have the potential to be used for forensic applications by associating successional changes with time to determine a PMSI. Two shifts in community composition at the first and second week were identified as common occurrences in both locations, even though differences in environmental factors outweighed successional changes. The influence of environmental factors, such as physical/chemical aspects of aquatic systems, indicates that the similar successional patterns are comprised of different communities. Future use of epinecrotic biofilms would need to consider geographic location, time of year, rainfall, and other conditions that can influence microbial survivorship. Further investigation into functional diversity may be more useful in utilizing this pattern in a universal manner to determine a PMSI. Community structure is related to function, and if the successional process of biofilm development remains similar, there may be consistent shifts in the functional profile regardless of the bacterial community composition. This functional profile is based on the idea of functional redundancy among species, which is when different species have the same role within an ecosystem. This relationship can be investigated using metagenomics and/or metatranscriptomics to determine if functionality of different bacterial species produces an identifiable and consistent pattern during succession.

The potential use of epilithic biofilms, or microbial communities that form on other substrates in streams and other flowing habitats, in aquatic biomonitoring was supported in this study. While the data presented here are an initial investigation into the variable microbial communities in flowing waters, they demonstrate that communities can be differentiated between substrate types, geographic location, and over time. This investigation provides initial information important for designing future studies to more explicitly evaluate how microbial community succession proceeds under a range of water quality conditions, and if specific communities or indicator species can be used for biomonitoring. Much like that for the epinecrotic communities, advances in amplicon-based and whole genome sequencing provide the tools for more comprehensively describing these microorganism communities under a range of conditions, and for identifying potential suites of species or genes that can be used as environmental indicators.
